# Beyond the Organic: A Biopsychosocial Analysis of Pediatric Functional Gastrointestinal Disorders—A Retrospective Chart Review

**DOI:** 10.3390/children13070885

**Published:** 2026-06-30

**Authors:** Julia Greuter, Margarete Bolten, Corinne Légeret

**Affiliations:** 1 Faculty of Medicine, University of Basel, 4056 Basel, Switzerland; 2 Switzerland Luzerner Psychiatrie AG Child & Adolescent Psychiatry, Children’s Hospital Central Switzerland (KidZ), 6004 Lucerne, Switzerland; 3 Department of Gastroenterology, University Children’s Hospital of Basel, 4056 Basel, Switzerland

**Keywords:** functional gastrointestinal disorders, paediatrics, biopsychosocial model, retrospective chart review, gut–brain interaction, hospitalization

## Abstract

**Highlights:**

**What are the main findings?**

- Pediatric functional gastrointestinal disorders (FGIDs) show stable prevalence over time, with no significant seasonal or yearly variation, supporting a chronic and non-seasonal disease pattern.
- Urbanicity is associated with higher FGID prevalence, suggesting an important role of environmental and contextual stressors in disease occurrence.
- Female adolescents show higher rates of pain-related FGIDs, while male adolescents more often require hospitalization and longer inpatient stays.
- FGIDs are strongly associated with healthcare burden, reflected in prolonged hospitalizations and the need for multidisciplinary care.

**What is the implication of the main finding?**

- Sex-specific and age-specific patterns in FGIDs highlight the need for early, interdisciplinary care pathways, including psychological screening-especially for adolescent girls, who face rising mental health burdens.
- Higher FGID prevalence in urban settings suggests environmental or psychosocial contributors, underscoring the importance of community-based prevention strategies and family centered interventions to reduce chronicity and unnecessary hospitalizations.

**Abstract:**

**Introduction:** Functional gastrointestinal disorders (FGIDs), conceptualized as disorders of gut–brain interaction, are among the most common chronic or recurrent conditions in childhood, affecting approximately 20–30% of children worldwide across community and clinical settings. FGIDs are associated with substantial impairments in quality of life, frequent school absences, and high levels of psychological comorbidity, contributing to a considerable burden for families and healthcare systems. Despite their high prevalence, the pathophysiology remains incompletely understood, with evidence pointing to a multifactorial interplay of biological, psychological, and environmental factors. Given their frequency across healthcare settings and their significant psychosocial and economic impact, a better characterization of FGIDs in real-world pediatric populations is needed. This retrospective chart review aimed to examine patterns of FGIDs and their associations with gender, temporal factors, geographic setting, and hospitalization burden in a Swiss pediatric cohort within a biopsychosocial framework. **Methods:** This retrospective chart review study included 1445 patients aged 0–18 years. Patients were selected based on having received an ICD-10 diagnosis attributed to FGID. The frequency and distribution of the aforementioned factors were determined, as well as their associations with each other. **Results:** A male predominance of FGIDs in newborns (*p* < 0.001), a female predominance in adolescents (*p* < 0.001), and sex-based differences in subtype distribution (*p* < 0.001) was found in this cohort of patients. A higher proportion of FGID cases were found among children in urban areas than in rural and suburban areas. Infants were hospitalized for significantly longer periods on average than older children and males were hospitalized for longer periods on average than females. **Discussion and Conclusions:** These findings highlight the importance of early, integrated, interdisciplinary care pathways. Given the growing mental health issues affecting adolescent girls and the well-documented bidirectional relationship between emotional stress and FGID symptoms, it is suggested that early psychological screening and family-based interventions could reduce the chronicity of symptoms, prevent unnecessary hospitalizations and improve long-term health outcomes.

## 1. Introduction

Functional gastrointestinal disorders (FGIDs) are among the most common health conditions in childhood. Clinically, FGIDs are recently redefined as disorders of gut–brain interaction, comprise a heterogeneous group of chronic or recurrent gastrointestinal symptoms that occur in the absence of structural or biochemical abnormalities [1,2]. Using the symptom-based Rome-IV criteria [3], contemporary systematic reviews show that FGIDs affect roughly one in five to one in three children worldwide, with pooled prevalence around 22% in both neonates/toddlers and children/adolescents in community samples [4,5]. In infants and toddlers, functional regurgitation, colic, dyschezia and constipation predominate, with cross-sectional prevalence between 24 and 35% in European and Turkish outpatient or well-baby settings [5,6]. Among school-aged children and adolescents, functional constipation, irritable bowel syndrome (IBS), functional abdominal pain disorders and functional dyspepsia are the leading diagnoses, with community prevalence estimates of any FGID ranging from about 10–30% in school surveys to nearly 25–30% in large national samples [4,7]. Clinical settings show an even greater concentration of these disorders: in tertiary gastroenterology clinics more than half to three-quarters of new referrals meet Rome criteria for at least one FGID [8], and in emergency departments the majority of non-organic abdominal pain and constipation visits appear functional in nature, generating long wait times and extensive investigations [9].

Although rarely life-threatening, pediatric FGIDs are associated with substantial health-related quality-of-life impairment for children and caregivers, often exceeding that seen in some organic gastrointestinal diseases. These disorders represent a significant burden not only for affected children but also for their families and the healthcare system. Children with FGIDs have lower physical and psychosocial functioning scores, frequent school absences, and high rates of anxiety, depression and other psychosocial stressors, embedded within a bidirectional gut–brain axis disturbance [1]. At the health-system level, FGIDs account for about 1 in 64 pediatric hospitalizations in the United States, with average stays of approximately 2–3 days and steadily increasing per-admission costs, particularly for constipation and abdominal pain that often prompt endoscopic procedures [10].

Despite the high prevalence rates and decades of research, the precise pathophysiology remains incompletely understood. Proposed mechanisms include genetic susceptibility, altered microbiota, visceral hypersensitivity, and neuroimmune and psychosocial factors [1,2]. This high prevalence across community, outpatient, emergency and inpatient settings, combined with significant psychosocial burden and rising healthcare utilization, underscores the need to better characterize FGIDs across pediatric care environments and to optimize timely, evidence-based, and resource-conscious management strategies.

Grounded in a biopsychosocial framework of pediatric FGIDs, this study aimed to characterize the distribution of cases and FGID subtypes across the study period. It further examined the associations of sex, temporal factors (season and year of diagnosis), and geographic setting (urban, small town, rural) with the frequency and subtype distribution of pediatric FGIDs. We hypothesized that both overall case numbers and subtype distributions would vary according to these factors. In addition, FGIDs were expected to impose a substantial hospitalization burden, with age and sex hypothesized to be significant predictors of length of hospital stay.

## 2. Materials and Methods

### 2.1. Design and Study Population

This study employed a retrospective chart review design using data extracted from the electronic medical record system of a Swiss tertiary care University children’s hospital. The study included all pediatric patients (aged 0–18 years) treated between 1 January 2022, and 31 December 2024 with the main diagnosis, meaning reason for treatment/hospitalization, of a FGIDs. Eligible participants were identified based on the presence of relevant diagnostic ICD-10 [11] codes recorded in their medical records. ICD-10 codes are assigned by the treating physician and used by the accounting department for billing purposes. Paediatric gastroenterologists use Rome IV criteria [3] in daily clinical practice and in studies to provide a more nuanced description of FGIDs, which are categorized into functional nausea and vomiting disorders, functional abdominal pain disorders, and functional defecation disorders. To enhance diagnostic specificity, ICD-10 diagnoses were mapped to their corresponding Rome IV categories where applicable. Table 1 provides an overview of the mapping between Rome IV and ICD-10 diagnoses in the study sample.

The study recorded patients who were treated for a FGIDs, therefore all patients with eligible ICD-10 codes were initially included, and all medical records were reviewed by one master student for accuracy and assigned the clinical ROM-IV criterion. Patients with incorrect ICD-10 codes were excluded. FGIDs identified were recorded as separate diagnoses and each new treatment for an FGIDs was recorded as a case. This means one patient can have more than one diagnosis and/or case. Exclusion criteria: Age above 18 years, FGIDs was not reason for treatment/hospitalization (was not main diagnosis). For an optimal display of patient data, clinical subgroups were founded: Feeding problems, motility problems, nausea/vomiting and pain (also in the sense of discomfort). The study group classified GERD under the ‘pain-related’ category—in the sense of discomfort—to clearly distinguish it from vomiting for the reader. The decision was made in accordance with the NASPGHAN/ESPGHAN guidelines [12], which delineate the general symptoms of GERD as discomfort/irritability and gastrointestinal symptoms, as heartburn/chest pain and epigastric pain.

In order to test the hypothesis that the frequency of FGIDs differs between urban and rural environment, postcodes were used and classified according to the Federal Office for Statistics in Switzerland (Bundesamt für Statistik, 2020 [13]), which divides postal codes based on the “Geographic Typologies, Urban/Town/Rural Typology, Status 2024.

### 2.2. Ethical Considerations

The study protocol was reviewed and approved by the local ethics committee (Ethikkommission Nordwestschweiz; project ID 2024-00796). All data were handled in accordance with applicable data protection regulations, and patient confidentiality was maintained throughout the study. No external funding was obtained.

### 2.3. Data Extraction

For all eligible patients, demographic and clinical data were extracted from the electronic medical records in a standardized manner to ensure consistency and completeness. Extracted variables included age, date of birth, sex, postal code, date of diagnosis, admission date, and discharge date.

Diagnoses were identified based on ICD-10 codes documented in the medical records. The assignment of ICD-10 diagnoses was performed by the treating physician. The hospital’s inpatient billing department calculated the mean costs during the study period for general paediatric patients (excluding oncology and intensive care patients, as they incur higher costs and would not be representative), which amounted to 2200 Swiss francs per day. The total hospital costs were calculated based on this amount.

### 2.4. Statistical Analysis

Statistical analyses were performed using IBM SPSS Statistics, version 29.0.2.0. Descriptive statistical analyses were conducted to summarize the demographic and clinical characteristics of the study population. Continuous variables are presented as means and standard deviations or medians and interquartile ranges, depending on the distribution of the data. Categorical variables are reported as frequencies and percentages.

Nonparametric chi-square tests of goodness of fit were used to examine the frequency of FGIDs across sexes and their distribution across different age groups. The same approach was applied to assess variations in FGID frequency according to seasonality, yearly trends, and geographic location. Normality of the length-of-stay data was assessed using both the Shapiro–Wilk test and visual inspection of Q-Q plots. Furthermore, a two-way analysis of variance (ANOVA) was performed to examine the association between sex, age group, and number of hospitalization days.

Post hoc comparisons were conducted using the Tukey honestly significant difference (HSD) test to further explore significant effects identified in the ANOVA.

A *p*-value of less than 0.05 was considered statistically significant for all analyses.

## 3. Results

### 3.1. Descriptive Data

A total of 1723 diagnoses from 1445 patients and 1611 cases were included in the analysis. The patients’ ages ranged from 0 to 18 years old. Fifty-one percent were male. The patients were divided into six age groups: newborns (ages 0–12 months), toddlers (ages 1–3), preschoolers (ages 4–5), primary schoolers (ages 6–12), teenagers (ages 13–17), and adults (age 18). Descriptives of the total sample are shown in Table 2.

The five most common diagnoses were feeding problems in newborns (n = 822; 47.7%), nausea and vomiting (n = 284; 16.5%), other and nonorganic constipation (n = 192; 11.1%), abdominal pain (n = 110; 6.4%), and gastroesophageal reflux without esophagitis (n = 110; 6.4%).

### 3.2. Sex Differences in FGID Frequency and Subtype Distribution

There was no significant difference in the overall distribution of FGID cases between male and female pediatric patients (χ^2^(1, n = 1445 patients) = 1.22, *p* = 0.269). However, subgroup analyses revealed age-specific sex differences.

In the neonatal subgroup, males were significantly overrepresented (χ^2^(1, n = 974) = 13.343, *p* < 0.001), whereas no significant sex differences were observed among toddlers, preschool children, and primary school children (*p* = 0.913, *p* = 0.423, and *p* = 0.494, respectively).

In contrast, female adolescents were significantly more prevalent than males (χ^2^(1, n = 153) = 19.77, *p* < 0.001).

The distribution of FGID subtypes differed significantly by sex (χ^2^(3, n = 1723) = 31.907, *p* < 0.001), with pain-related FGIDs being more common among females and feeding-related FGIDs more frequent among males. Detailed frequencies and percentages are presented in Table 3.

### 3.3. Seasonal Patterns in FGID Frequency and Subtypes

No statistically significant differences in FGID case numbers were observed across seasons (χ^2^(n = 1611 cases) = 5.206, *p* = 0.157) or across the three years of data collection (2022–2024) (χ^2^(n = 1611) = 1.579, *p* = 0.454). Similarly, there was no significant association between FGID subtype distribution season or year of data collection, indicating stable incidence rates for FGID over time.

### 3.4. Environmental Differences in FGID Frequency and Subtypes

Significant differences were found between urban and rural areas in relation to FGID case frequency (χ^2^(2) = 54.66, *p* < 0.001), with a higher proportion of cases observed in urban areas compared to small-town or rural regions.

However, FGID subtype distribution did not significantly differ between environmental settings (χ^2^(6) = 12.182, *p* = 0.058), suggesting comparable clinical presentations across regions.

### 3.5. Hospitalization Burden

For all groups, the Shapiro–Wilk test indicated that the data did not significantly deviate from normality, and the Q-Q plots showed no substantial departures from a normal distribution. Therefore, the normality assumption required for ANOVA was considered met.

After exclusion of outliers (160 outliers have been excluded, therefore the calculation is based on 1451 cases) based on the 1.5 IQR criterion, the mean length of hospital stay was 7.64 days over all FGID cases to treat the functional problem. The total number of hospitalization days over the three-year period amounted to 11,085 days. Based on an average daily cost of 2200 CHF (average DRG cost/day for the University Children’s Hospital of Basel, Switzerland), this corresponds to a total estimated cost of 24,387,000 CHF, indicating a substantial healthcare burden caused with pediatric FGIDs.

**Figure 1:**
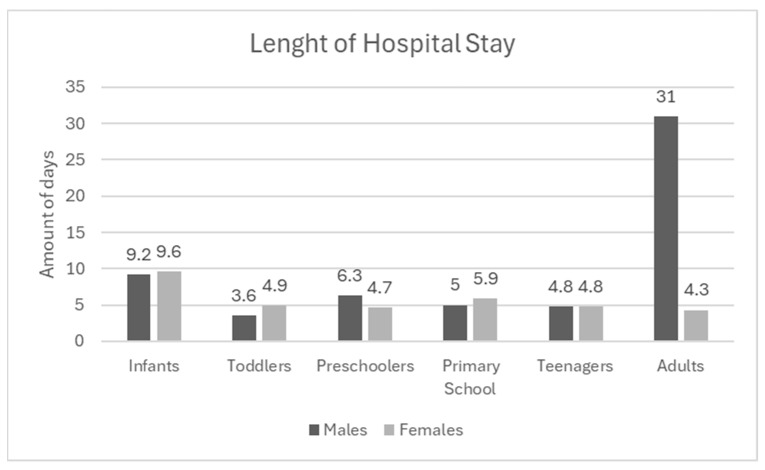
Hospital duration by age group and sex.

Statistical analysis revealed that age group and sex were significant predictors of hospitalization duration (*p* = 0.047). However, these variables explained only 7.2% of total variance in the length of hospital stay (R^2^ = 0.072). Infants had significantly longer hospital stays compared to toddlers, preschool children, primary school children, and adolescents (*p* < 0.001). In addition, males had longer hospital stays than females on average (*p* = 0.006) (Figure 1).

## 4. Discussion

This retrospective chart review provides clinically relevant insights into the epidemiology, clinical presentation, and healthcare burden of pediatric functional gastrointestinal disorders (FGIDs) within a biopsychosocial framework. Based on a large Swiss sample of over 1400 pediatric patients, several key findings emerged.

First, the absence of significant seasonal or yearly variation suggests that FGIDs in childhood represent relatively stable conditions that are largely independent of short-term environmental fluctuations. This stability likely reflects the predominance of enduring individual and familial factors—such as gut–brain axis dysregulation, temperament, and family functioning—over transient external influences. It may also indicate consistent diagnostic practices and stable healthcare access across the study period. While early work by Saps et al. [14] reported pronounced seasonal patterns, attributed to increasing academic stress toward the end of the school term, our findings did not replicate these effects. Instead, only minor fluctuations were observed, with slightly higher presentation rates in spring and autumn.

These findings align more closely with studies emphasizing psychological rather than seasonal mechanisms. Pollard et al. [15] demonstrated that improvements in FGID symptoms over the summer were closely associated with reductions in anxiety and improved sleep, rather than changes in diet or physical activity. Similarly, studies from Saudi Arabia [16] and Spain [17] reported minimal or no seasonal variation. Together, these data suggest that FGID symptomatology in pediatric clinical populations is not primarily driven by seasonality, but rather by fluctuations in psychological stress. Clinically, this underscores the importance of routinely assessing anxiety, stress, and family context throughout the year, rather than focusing on presumed “high-risk” periods.

Second, no overall gender differences in FGID prevalence were observed, consistent with pediatric data from India [7] and Sweden [18]. Moreover, stress has been identified as a key predictor of FGIDs in adolescents regardless of gender [19]. Nevertheless, important age- and symptom-specific patterns emerged. In our cohort, adolescent girls showed a higher prevalence of FGIDs, particularly pain-related subtypes, and reported greater symptom burden. This finding aligns with evidence linking female sex to increased visceral sensitivity and a higher prevalence of affective comorbidities [20]. Consistent patterns have been reported in adult populations [21], pediatric samples from Jordan [22], and in a meta-analysis across Europe, South America, and Asia [23]. Proposed mechanisms include sex-specific differences in gastrointestinal physiology, such as slower gastric emptying and colonic transit, increased risk of post-infectious irritable bowel syndrome, and greater activation of affective and autonomic brain regions in response to visceral stimuli [24].

Psychological factors likely further contribute to these differences. Depressive symptoms and anxiety are more prevalent in females and are known to exacerbate FGID symptoms [25]. A growing body of evidence indicates that adolescent girls are disproportionately affected by internalizing disorders [26]. This trend has intensified in recent years: Swiss national data show a marked increase in psychiatric hospitalizations among females aged 10–24 years between 2020 and 2021, particularly for depression and suicide-related outcomes (Swiss Federal Statistical Office [27]), consistent with international findings following the COVID-19 pandemic [28,29]. Together, these findings suggest that sex differences in FGIDs are likely mediated by an interplay of biological and psychosocial mechanisms.

A notable and unexpected finding was the predominance of male infants among FGID cases, particularly those presenting with feeding difficulties. This contrasts with existing literature, which generally reports no gender differences in early childhood FGIDs [5]. Large cohort data have instead identified perinatal factors such as birth weight, cord blood pH, and maternal age as key predictors [30], variables that were not available in our dataset. Only limited prior evidence suggests a similar male predominance in feeding difficulties, without a clear biological explanation.

Psychosocial mechanisms may therefore be particularly relevant. Established risk factors for feeding disorders include parental inexperience (e.g., firstborn status) and maternal depression [31]. Interestingly, emerging evidence suggests that male offspring may be associated with an increased risk of maternal postnatal depression [32,33], potentially mediated by inflammatory processes related to pregnancy complications such as pre-eclampsia [34,35]. In this context, the observed male predominance in infants may reflect early interactional disturbances rather than infant-specific vulnerability. However, this interpretation remains speculative and highlights the need for future studies incorporating perinatal and psychosocial variables.

Third, the significantly higher proportion of cases in urban areas highlights the importance of contextual environmental factors, or urbanicity. The data presented is considered representative of the subject matter due to the exclusive provision of paediatric care in tertiary care centres in Switzerland. This ensures the collection of unbiased data relevant to the specified topic. Urban living may be associated with increased exposure to psychosocial stressors, lifestyle-related risks, and environmental influences, as well as differences in healthcare utilization and parental help-seeking behavior. Notably, the absence of differences in FGID subtype distribution between urban and rural areas suggests that, while urbanicity may influence overall prevalence, the specific clinical manifestation of FGIDs is primarily shaped by shared underlying mechanisms and individual-level factors. Evidence on urban–rural differences in pediatric FGIDs remains scarce and inconsistent; for example, Huang et al. [36] reported higher prevalence rates in rural Chinese populations. These discrepancies highlight the need for further research into contextual determinants.

Finally, the substantial number of hospitalization days underscores the considerable healthcare burden associated with pediatric FGIDs. Prolonged hospital stays likely reflect the complexity of these disorders, including the need to exclude organic disease and the involvement of multidisciplinary care. Age and sex were significant predictors of length of stay, although they explained only a small proportion of the variance. An American [37] review compared FGIDS cases between 1997 and 2009, revealing a consistent hospital stay of 2 days, but an increase in costs from $6115 to $18,000 per case in 2009. Longer hospitalizations in infants may be attributable to increased medical vulnerability, limited communicative abilities, and a more cautious diagnostic approach [38].

Overall, these findings further support a biopsychosocial model in which contextual factors influence prevalence, while individual and psychosocial variables are more critical in shaping disease course and healthcare utilization.

Limitations of the study include its retrospective nature. To ensure the most consistent assignment of ICD-10 codes possible, this task was performed by a single person; however, this meant that no reliability check was conducted—another limitation of this data set. It is also important to acknowledge that a single patient may possess multiple diagnoses and/or cases within our dataset, which in turn imposes limitations on the statistical methods that can be employed, particularly chi-square analyses.

In summary, our findings support a biopsychosocial model in which relatively stable individual vulnerabilities interact with dynamic psychological and contextual factors. While macro-level variables such as urbanicity appear to influence overall prevalence, individual and psychosocial factors are more critical in determining symptom expression, disease course, and healthcare utilization.

## 5. Conclusions

This retrospective chart review provides clinically relevant insights into the presentation and management of pediatric functional gastrointestinal disorders (FGIDs) in a large Swiss sample. The frequent co-occurrence of FGIDs and psychological distress underscores the need to conceptualize these conditions not in isolation, but as interconnected manifestations of dysregulation within the gut–brain axis. The higher prevalence of FGIDs in urban areas may reflect increased exposure to psychosocial stressors and lifestyle-related risk factors associated with urbanicity.

Taken together, these findings highlight the need for early, integrated, and interdisciplinary care pathways. FGIDs in children and adolescents should be managed as biopsychosocial conditions requiring nuanced, context-sensitive, and gender-informed approaches. Given the rising mental health burden among adolescent girls and the strong interplay between emotional stress and FGID symptoms, close collaboration between pediatricians, child and adolescent psychiatrists, psychologists, and allied health professionals is essential from the outset. Early psychosocial screening and family-based interventions may help reduce chronicity, prevent unnecessary hospitalizations, and improve long-term outcomes [39,40].

Future research should further disentangle the relative contributions of environmental, psychological, and biological factors to symptom development and persistence. Longitudinal studies with repeated assessments of anxiety, stress, sleep, and school-related burden across the year—ideally combined with physiological markers of gut–brain axis functioning—may help clarify why some children exhibit fluctuating symptom patterns while others remain stable. 

## Figures and Tables

**Table 1:** Mapping of Rome IV and ICD-10 Diagnoses in the Study Sample.

Age Group	Rome-IV Diagnose	ICD-10 Code	
Infants & Toddlers	G1 Infant regurgitation G2 Infant rumination syndrome	P92.1	Regurgitation/ rumination in newborn
	G3 Cyclic vomiting syndrome	P92.0	Vomiting in newborn
Children & Adolescents	**H1. *Functional nausea and vomiting disorders***		
	**H1b.** *Functional nausea and functional vomiting*	R11	Nausea and vomiting
	**H1a.** *Cyclic vomiting syndrome*	F50.5	Vomiting associated with other psychological disturbances
	**H2a.** *Functional dyspepsia*	K30 R10.1	Functional dyspepsia Upper abdominal pain
	**H2. *Functional abdominal pain disorders***		
	**H2b.** *Irritable bowel syndrome*	K58.3	Irritable bowel syndrome with alternating (mixed) bowel habits
		K58.8	Other and unspecified irritable bowel syndrome
		R19.4	Change in bowel habit
	**H2d.** *Functional abdominal pain—not otherwise specified*	R10.3 R10.4 K59.8 K59.9 F45.32	Lower abdominal pain Unspecified abdominal pain Other functional intestinal disorder Functional intestinal disorder unspecified Somatoform disorder lower GIT
	**H3. *Functional defecation disorders***		
	**H3a.** *Functional constipation*	K59	Constipation unspecified
	**H3b.** *Nonretentive fecal incontinence*	F98.1	Nonorganic encopresis
Infants and Toddlers	No clear equivalent under Rome IV	K21.9 P92.2 P92.3 P92.5 P92.8 F98.2	GERD without esophagitis Slow feeding newborn Underfeeding newborn Breastfeeding difficulty Other feeding problems newborn Feeding disorder infancy/childhood
	No clear equivalent under Rome IV	K59.4	Anal spasm
		R10.2	Pelvic/perineal pain

**Table 2:** FGID case numbers in different age groups.

Age Group	Ages	Male	Female	Total
Newborns	0–12 months	544	430	974
Toddlers	1–3 years	42	41	83
Preschool Children	4–5 years	25	31	56
Primary School Children	6–12 years	82	91	173
Teenagers	13–17 years	49	104	153
Adults	18 years	1	5	6
				1445

**Table 3:** Frequencies of FGID Subgroups.

	Male	Female	Total Frequency	Percent %
Pain-Related	110	179	290	16.8
Motility/Defecation	120	133	253	14.7
Nausea/Vomiting	124	161	285	16.5
Feeding	497	399	896	52.0
Total	852	872	1723	100.0

## Data Availability

The dataset is available on request from the corresponding author.

## Abbreviation

The following abbreviation is used in this manuscript:

FGIDs	Functional gastrointestinal disorders
